# Spontaneous spinal epidural hematoma of the thoracic spine after herbal medicine: a case report

**DOI:** 10.1186/s12906-018-2354-y

**Published:** 2018-10-29

**Authors:** Eo Jin Kim, Joonghyun Ahn, Seung-Ju Kim

**Affiliations:** 1 Department of Orthopaedics, Hanil General Hospital, 308 Uicheon-ro, Dobong-Gu, Seoul, 132-703 South Korea; 2 Department of Orthopaedics, CM Chungmu Hospital, Yeongdeungpo-go, Seoul, 150-034 South Korea

**Keywords:** Spontaneous spinal epidural hematoma, Surgical treatment, Herbal medicines, Black garlic, Spinal cord

## Abstract

**Background:**

Spontaneous spinal epidural hematoma (SSEH) is an uncommon disease, but it can lead to acute cord compression with disabling consequences. Identifiable reasons for spontaneous hemorrhage are vascular malformations and bleeding disorders. However, SSEH after taking herbal medicines has not been described yet.

**Case presentation:**

A 60-year-old female experienced sudden back pain combined with numbness and weakness in the lower limbs for several hours with no trauma, drug use, family history or any disease history. Her deep tendon reflexes were normoactive, and Babinski was negative. An emergent MRI showed a spinal epidural hematoma extending from T3 to T5. She was taken to surgery after immediate clinical and laboratory evaluations had been completed. Emergency decompression with laminectomy was performed and the patient recovered immediately after the surgery. Additional history taken from the patient at outpatient clinic after discharge revealed that she had been continuously taking herbal medicine containing black garlic for 8 weeks.

**Conclusion:**

To our knowledge, no report has been previously issued on SSEH after taking herbal medicines. Although contradictory evidence is present on bleeding risks with herbal uses, we believe that it’s reasonable to ascertain if patients with SSEP are taking herbal medication before or during spinal surgery.

## Background

Spinal epidural hematoma (SEH) is an idiopathic aggregation of blood in epidural space which can be as acute, chronic, spontaneously, post traumatic, or iatrogenic [1]. SEH occurring without a trauma is called as spontaneous SEH (SSEH) and it is an uncommon neurosurgical emergency which can lead to acute cord compression with disabling consequences [2]. The incidence of SSEH was estimated to be 0.1 patients per 100,000 populations per year [3]. As SSEH is one of the potentially reversible pressure lesions on the spinal cord and roots, its prompt diagnosis and treatment have a vital importance [1]. Although some nonsurgical treatments have been reported in cases that neurologic deficit improves in the early phase of disease [1], early surgical decompression (Laminectomy) is the first-line treatment modality for SSEH [4, 5].

The etiology of SSEH remains unknown; however, some predisposing factors have been reported, including long term aspirin use as platelet aggregation inhibitor, anticoagulant therapy for prosthetic cardiac valves, therapeutic thrombolysis for acute myocardial infarction, hemophilia, factor XI deficiency, vascular malformation, Paget’s disease and pregnancy [6–8]. However, to the best of our knowledge, no report has been previously issued on SSEH after taking herbal medicines. Here, we report a case of a 60-year-old woman who presented with SSEH after taking herbal medicines and treated successfully by surgical decompression.

## Case presentation

**Fig. 1:**
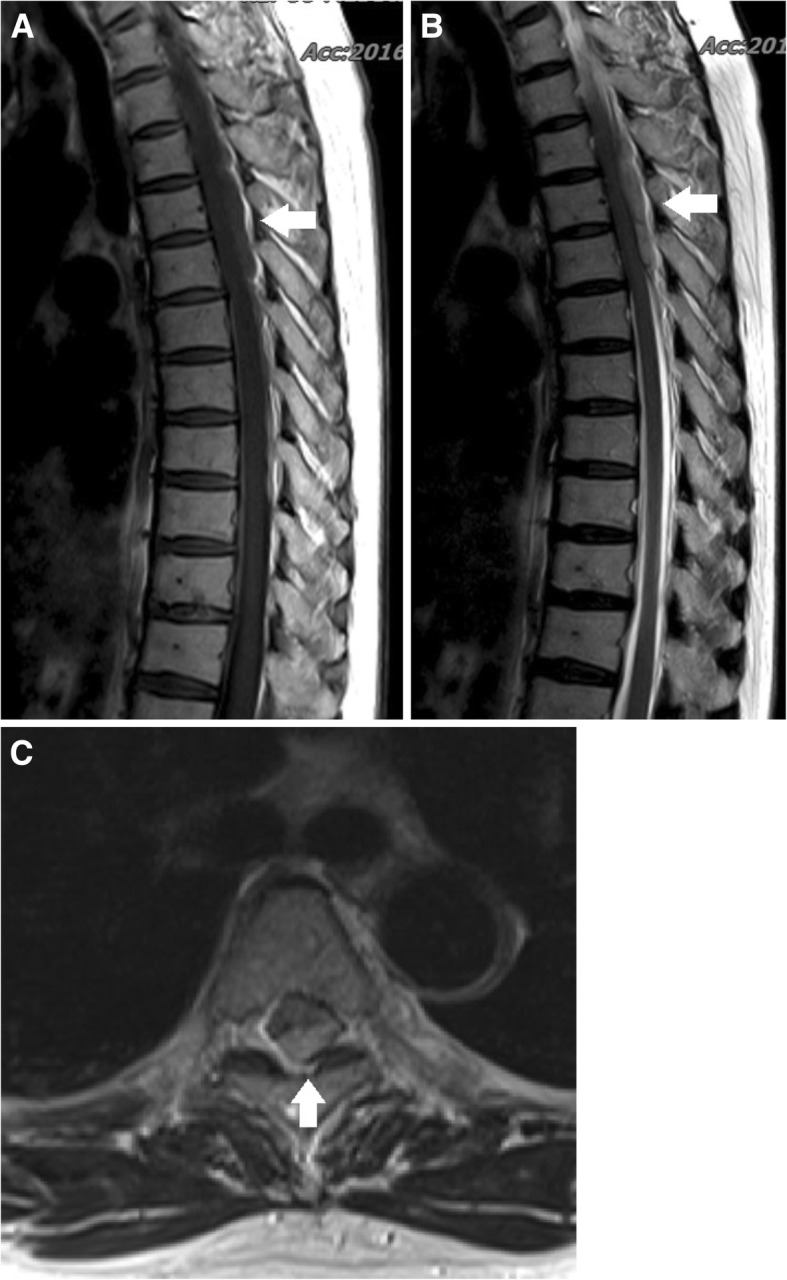
(**a**) Preoperative T1-weighted MRI shows iso signal intensity posterior spinal epidural mass lesion (sagittal image) (**b**) Preoperative T2-weighted MRI shows high signal intensity posterior spinal epidural mass lesion(sagittal image). (**c**) Axial image shows epidural hematoma on the dorsal portion of spinal canal (T2-weighted MRI)

**Fig. 2:**
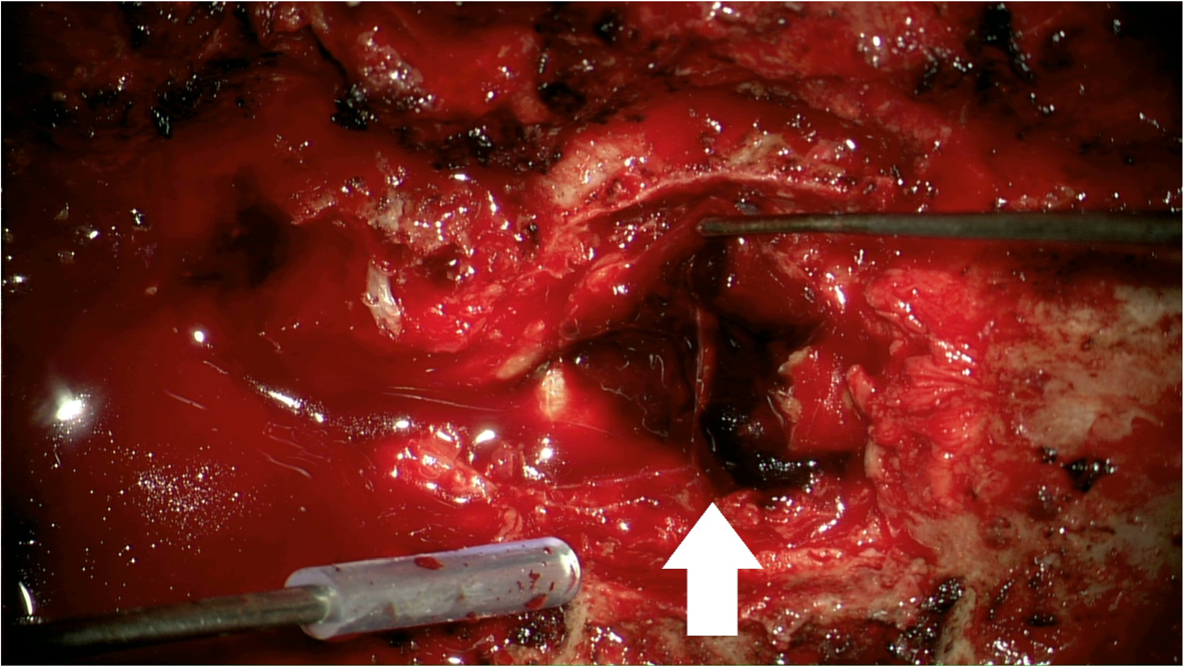
Total laminectomy from T3 to T5 and evacuation of epidural hematoma (red-colored mass) was performed

**Fig. 3:**
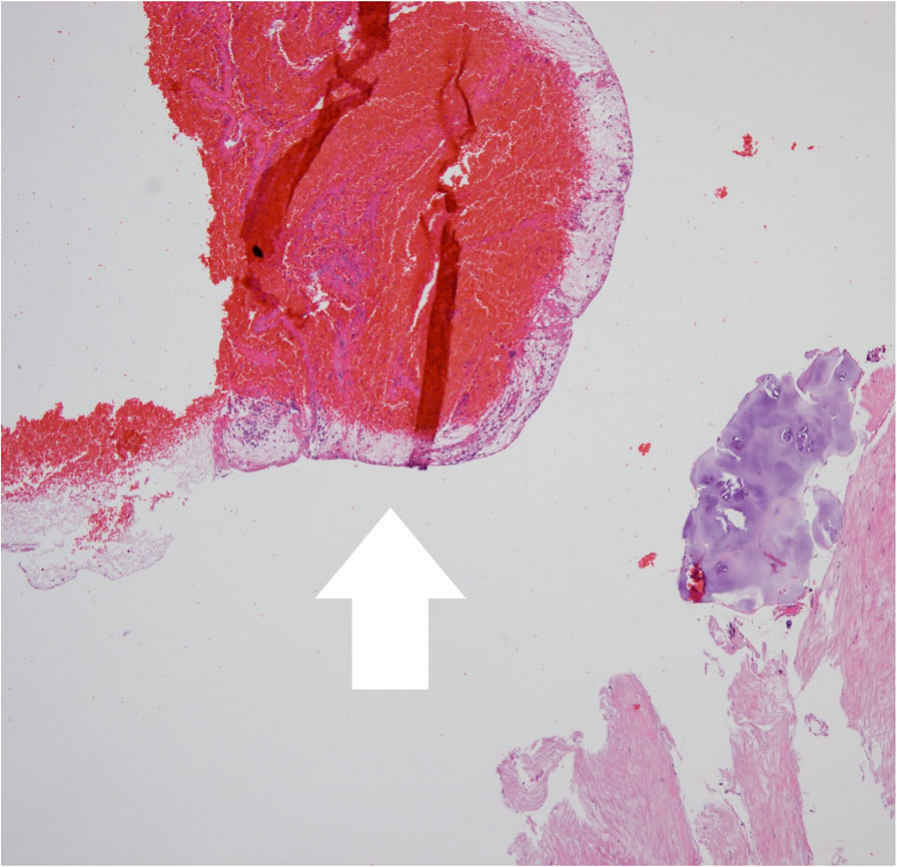
Hematoxylin and eosin stain, 40×. Photomicrograph shows a lot of red blood cells

A 60-year healthy female who was a hospital janitor of our institute visited an emergency department with a one-hour history of sudden low back pain with lower extremity motor and sensory deficit aggravated by bending. In her medical history, there was no recent trauma, no familial bleeding disorder, or no anticoagulation treatment. The patient’s previous surgical history revealed an open discectomy procedure at L3 to L4 due to herniated intervertebral disc in 2011. The neurologic exam revealed complete paralysis of the bilateral lower extremities and the symmetrical disappearance of body sensation below the T7 dermatome. On MRI, posterior to the spinal cord, there was a mass lesion in the epidural space at T3–T5 levels, which was isointense on T1- weighted images and hyperintense on T2 weighted images compared to spinal cord intensity, consistent with acute hematoma (Fig. 1). Comprehensive conservative treatment failed to improve her symptoms. The patient underwent emergency surgical treatment within 24 h after initial onset of symptoms. Total laminectomy from T3 to T5 was performed, and blood clot located at the dorsal portion of the spinal cord was evacuated (Fig. 2). Upper and lower spinal levels were clear for any additional presence of hematoma mass. Microscopic histological examination of the resected mass revealed a hematoma(Fig. 3). The patient showed dramatic improvement after surgery. The patient discharged without any motor or sensory impairment after 3 weeks and returned to work after a month. Additional history taken from the patient at outpatient clinic after discharge revealed that the patient did not have any positive past medical history but had been continuously taking herbal medicine containing black garlic for 8 weeks.

## Discussion and conclusions

Identifiable underlying disorders of SSEH beside trauma are bleeding disorders, spinal infections, spinal tumors, spinal interventions, and vascular malformations [6, 9]. Herbal remedies are used to treat a large variety of diseases in Asian countries. However, a number of herbal preparations have been reported to cause variations in clotting time by inhibition of coagulation factors or platelet activity [10]. Interestingly, several herbal medicines including garlic, feverfew, ginger, and ginseng have been associated with potential increased bleeding [11, 12].

According to a recent study [13], it has been reported that it seems prudent to stop taking high dosages of garlic seven to 10 days before surgery because garlic can prolong bleeding time. Beckert et al. [14] reported that an increased risk of bleeding, substantiated by anecdotal reports, has been attributed to the use of certain herbs, and numerous in vitro experiments have identified some herbal extracts as platelet inhibitors. Tsai et al. [15] demonstrated that for those patients who are taking conventional anti-clotting medications with herbal medicines for cardiovascular diseases, the potential risks of increased bleeding due to drug-herbal medicine interactions should not be ignored. Nevertheless, the risks attributed to herbal medicines are often ignored or underestimated. Questions about herbal medicines are not routinely asked in clinics. In addition, as many of these herbal supplements are available over the counter, many patients do not disclose these when listing medications to health care providers. We can’t comment on all the specific herbal medicines used, because herbs contain hundreds of chemical constituents, some are more pharmacologically active than others. As plants contain many different kinds of compounds, they usually have multiple actions.

In our case, the patient didn’t have an abnormal bleeding tendency and any positive past medical history on admission. Preoperatively, laboratory tests such as PT (prothrombin time) or aPTT (activated partial thromboplastin time) were normal range. However, bleeding time was slightly prolonged to 8 min (normal range, 2 to 6 min) which was not noticed before surgery, and the patient had taken garlic for 2 months. It has been reported that garlic can inhibit platelet aggregation, and the bleeding time increased in the patients who had received garlic [9, 16, 17]. This bleeding tendency is one of predisposing factors of SSEH and may cause the disease [8, 9]. Although contradictory evidence is present on bleeding risks with herbal uses, we believe that it’s reasonable to ascertain if patients are taking herbal medication, especially in Asian population, and to discontinue any herbal preparations before undergoing surgical procedures if it is not inevitable.

In conclusion, we report a patient with SSEH after taking herbal medicine. As side-effects and herb–drug interactions could be significant or fatal, we believe that a detailed drug history including herbal remedy use should be taken by physicians before or during surgery especially in patients with SSEH.

## Abbreviation

SSEH: Spontaneous spinal epidural hematoma
